# Afebrile benign convulsions with or without a reversible splenial lesion in two pediatric patients with COVID-19

**DOI:** 10.1186/s12887-023-04025-x

**Published:** 2023-04-26

**Authors:** Yun Young Lee, Young Ok Kim

**Affiliations:** 1 Department of Radiology, Chonnam National University Children’s Hospital, Gwangju, Republic of Korea; 2 Department of Pediatrics, Chonnam National University Children’s Hospital, Gwangju, Republic of Korea; 3 Department of Pediatrics, Chonnam National University Medical School, Gwangju, Republic of Korea

**Keywords:** COVID-19, Seizures, Reversible splenial lesion, Child, Infant

## Abstract

**Background:**

Seizures in children with coronavirus disease 2019 (COVID-19) were markedly increased during the Omicron variant surge. Most seizures occurred with fever. New-onset afebrile seizures were rarely reported; therefore, their courses are not well-known.

**Case presentation:**

Two patients (7 and 26 months of age, respectively) with COVID-19 showed recurrent afebrile seizures immediately after resolution of a fever lasting for 2–3 days. Bilateral convulsive seizures lasted for approximately 1 min/episode (6 of 7 total episodes) and occurred 3–4 times within 2–3 h. However, the patients were alert between seizures, which is in contrast to seizures occurring with encephalopathy or encephalitis. Only one episode required acute antiseizure medication. Brain magnetic resonance imaging showed a reversible splenial lesion in one patient. The serum uric acid level was slightly increased (7.8 mg/dL) in this patient. Electroencephalography findings were all normal. During the follow-up period, no seizures or developmental problems have been observed.

**Conclusions:**

COVID-19-associated, afebrile benign convulsions with or without a reversible splenial lesion are similar to ‘benign convulsions with mild gastroenteritis’; therefore, continuation of antiseizure medication does not seem necessary.

## Background

Children with coronavirus disease 2019 (COVID-19) present with diverse neurologic symptoms or signs [1–9]. Specific neurologic issues, such as seizures, meningeal irritation, and mental changes, have been reported more frequently in more critically ill patients [3, 8, 9]. Neurologic diseases reported in pediatric patients with COVID-19 include convulsive diseases, encephalitis or encephalopathy, demyelinating diseases, cerebral ischemic or hemorrhagic stroke, and cranial nerve palsy [1–8]. The proportion of seizures among all children admitted to the hospital with COVID-19 was estimated to be < 10% before the Omicron variant surge but increased up to 20% after the outbreak [8, 10–13]. Before the Omicron variant surge, most seizures were reported not as isolated diseases but as additional signs in a febrile period associated with other major inflammatory diseases [3, 7]. Only Kurd et al. have reported that seizures were the main manifestation during the early stages of COVID-19 in some children (6.2% of 175 children visiting an emergency department) [4]. During the Omicron variant surge in early 2022, approximately 69–85% of seizures were reported as febrile convulsions or provoked seizures during a febrile period [10–12]. New-onset, afebrile seizures in previously healthy pediatric patients with COVID-19 are uncommon, especially those with a reversible splenial lesion. In addition, their treatment and prognosis are rarely described in detail. Here, we reported two patients with afebrile benign convulsions associated with COVID-19 during the Omicron variant outbreak in South Korea (between March and April 2022). One of the patients had a reversible splenial lesion, which is rarely reported in patients with COVID-19; in particular, there are no reports of such lesions in those without encephalopathy. Their clinical characteristics were similar to those observed in young children with benign convulsions with mild gastroenteritis (CwG) due to rotavirus or norovirus [14–18].

## Case presentation

### Case 1

A 7-month-old male infant with COVID-19 visited the emergency room (ER) due to new-onset recurrent seizures without a fever. Before seizure onset, he had experienced a fever, cough, and intermittent vomiting for three days. He was diagnosed with COVID-19 *via* polymerase chain reaction (PCR) sequencing of nasal and throat swabs on the second febrile day. Prior to his diagnosis, his mother was diagnosed with COVID-19. On the 4th day when the fever had subsided, seizures suddenly developed. This patient was previously healthy, had an uneventful birth at a gestational age of 38 weeks and weighing 3,580 g, and had unrelated parents. No family members had seizures or developmental problems.

He had brief bilateral tonic-clonic seizures (4 episodes within 2 h). Each episode lasted for approximately 1 min and was associated with upward eyeball deviation, crying, and facial flushing. There was no change in his mental state between episodes, in contrast to cases of encephalopathy or encephalitis. Brain computed tomography, electrocardiography (EKG), chest roentgenography, and basic laboratory tests including measurement of serum glucose, electrolytes, and uric acid levels were all normal. Brain magnetic resonance imaging (MRI) was not available for patients with mild symptoms at that time. The patient had no more seizures or fever in a negative-pressure isolation room; therefore, he was discharged that day. At follow-up 10 days later in the outpatient clinic, his growth and development were normal. Brain MRI and electroencephalography (EEG) were performed within 30 days of the onset of his first seizure. The results were all normal (Table 1). During the one-year follow-up period, no seizures or developmental problems were observed.

**Table 1:** Characteristics of patients with COVID-19-associated afebrile benign convulsions

	Patient 1	Patient 2
Age (sex)	7 months (male)	26 months (male)
Development	Normal	Normal
Underlying diseases	No	No
Gestational age at birth	38 weeks	37 weeks
Previous history of seizures	No	No
Family history of seizures	No	No
First symptoms	Fever	Fever
Fever duration	3 days	2 days
COVID-19 diagnosis by PCR (date^a^)	2nd	1st
Respiratory symptoms	Cough	No
Enteric symptoms	Vomiting	No
Mental state	Alert	Alert
Seizure characteristics (afebrile)		
Onset (date^a^)	4th	3rd
Number	4	3
Duration per episode	1 min (all)	1 min (1st & 2nd ), 10 min (3rd )
Interval from the first to the last seizure	2 h	3 h
Type	Bilateral TC	Bilateral T or TC
Antiseizure treatment (transient)	No	Lorazepam, fosphenytoin
Recurrence of seizures during follow-up	No	No
Developmental problems during follow-up	No	No
Brain MRI (days from seizure onset)	Normal (30 days later)	Reversible splenial lesion (the same day)
Electroencephalography (days from seizure onset)	Normal (15 days later)	Normal (47 days later)
Laboratory findings at seizure onset^c^		
WBC count (/µL)	15,100	11,600
Serum uric acid (mg/dL)	4.7	7.8
Serum glucose and electrolytes	Within normal limits	Within normal limits
Serum C-reactive protein (mg/dL)	0.27	< 0.01

Abbreviation: COVID-19, coronavirus disease 2019; PCR, polymerase chain reaction; TC, tonic-clonic; T, tonic; MRI, magnetic resonance imaging; WBC, white blood cell

^a^The date was counted from the onset of the first symptom

### Case 2

A 26-month-old male patient with COVID-19 was transferred to the ER due to two events of new-onset brief tonic seizures without a fever (approximately 1 min/episode). Prior to his seizures, he and his family members were diagnosed with COVID-19. COVID-19 was confirmed on the first febrile day *via* PCR sequencing using samples from his nose and throat. He had been febrile for the previous two days, but was not febrile on the 3rd day when his seizures developed. He was previously healthy with normal development and growth. He was born without any specific perinatal events at a gestational age of 37 weeks from unrelated healthy parents and weighed 3,000 g. There was no history of seizures or developmental disorders in his family.

**Fig. 1:**
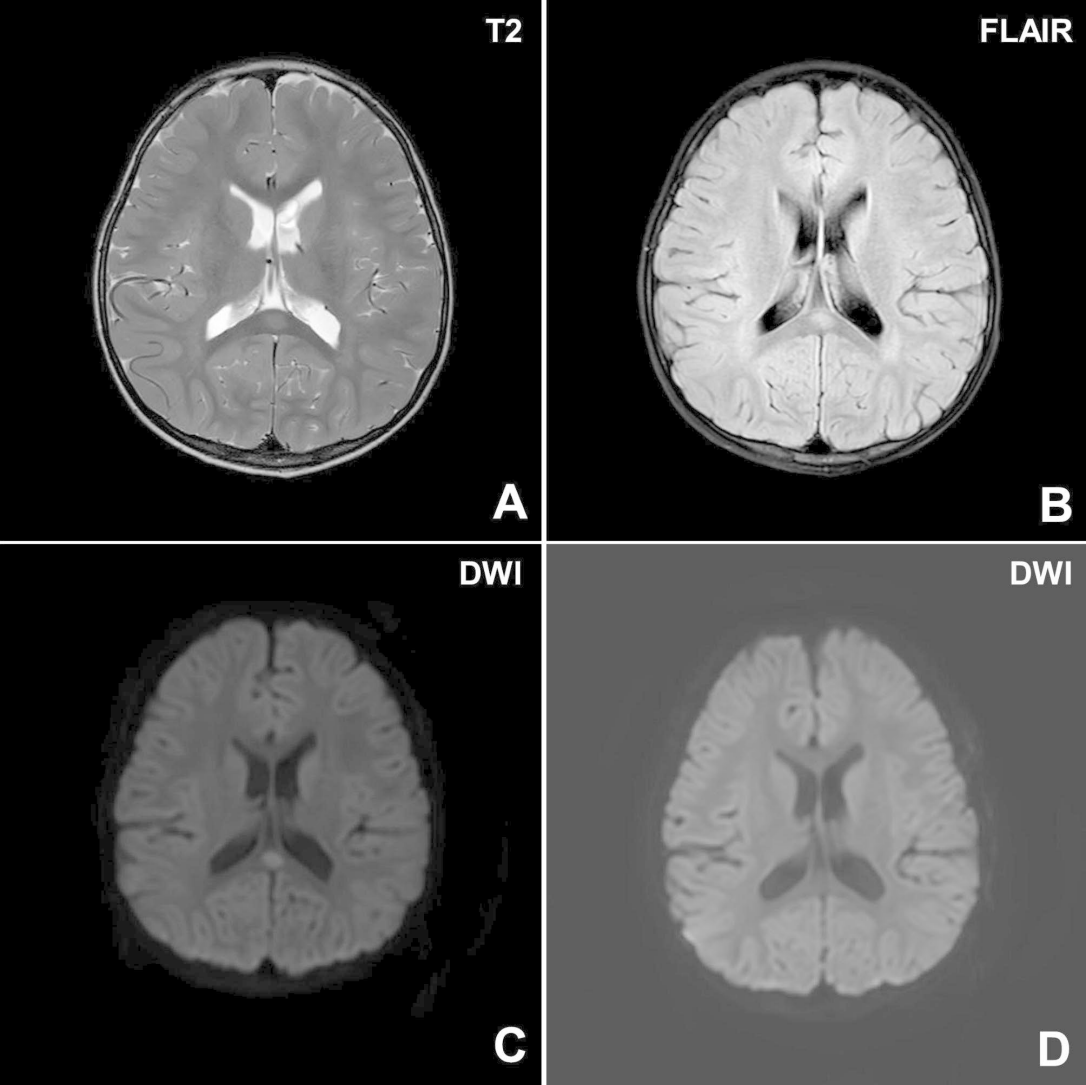
Brain magnetic resonance imaging (MRI) at the time of diagnosis (**A–C**) and 47 days after the first visit (**D**) in patient 2. Axial T2-weighted (**A**) and fluid-attenuated inversion recovery (FLAIR, B) images showed a 1-cm hyperintense lesion in the splenium of the corpus callosum. This lesion with diffusion restriction (**C**) on diffusion imaging at the time of diagnosis was not detected in the corpus callosal splenium 47 days after the first visit (**D**)

His third seizure was observed three hours after the first seizure onset in the ER; it was a bilateral tonic-clonic seizure and lasted for 10 min even with two injections of lorazepam. However, fosphenytoin administration was successful at controlling the seizure. Although the patient briefly lost awareness during the seizures, he became alert between and after seizures, in contrast to seizures associated with encephalopathy. He recovered quickly even after antiseizure medication. Chest roentgenography, EKG, and most basic laboratory tests performed in the ER were all normal, except for a slightly increased serum uric acid level (7.8 mg/dL). Brain MRI on admission showed a 1-cm T2-hyperintense lesion with diffusion restriction in the splenium of the corpus callosum (Fig. 1. A–C). His general condition was relatively good without seizures or a fever in a negative-pressure isolation room; therefore, he was discharged the next day without medication. At a follow-up visit, brain diffusion-weighted MRI that was performed 47 days later revealed no nodular diffusion-restricted lesions in the corpus callosal splenium (Fig. 1. D). An EEG performed during sleep on the same day was normal (Table 1). During the one-year follow-up period, seizures did not recur. The patient developed normally.

## Discussion and conclusions

The two children with COVID-19 described in this report presented with recurrent, brief, afebrile convulsions. They visited our hospital during the Omicron variant waves; however, further laboratory confirmation of the Omicron variant was not available commercially. They were aged 7 and 26 months, respectively, and were previously healthy with normal development. They had no family history of seizures. Seizures appeared immediately after the fever had subsided. Neither patient had mental confusion, which is in contrast to patients with encephalopathy or encephalitis. Seizures occurred 3–4 times within 2–3 h. Six out of seven seizures lasted for approximately 1 min. Only one episode lasted for 10 min and responded to fosphenytoin but not to lorazepam. The seizures were bilateral tonic-clonic or tonic. One patient showed a reversible splenial lesion on brain MRI, which has not yet been reported in patients with COVID-19 without encephalopathy. The serum uric acid level was slightly elevated in this patient (7.8 mg/dL). In the other patient, brain MRI was not available on admission. EEGs were also not available for both of these patients on admission because they were available only for critically-ill patients with COVID-19 in the intensive care unit at that time. EEGs that were performed later were all normal. These new-onset stereotyped seizures in young children showed a benign course without concomitant major infectious/inflammatory symptoms or neurologic deficits. These phenotypes and disease course were quite similar to those of CwG [14–18].

CwG occur sporadically in previously healthy infants and young children (typical range, 6–36 months of age) [14, 15]. Afebrile short-lasting and recurrent seizures typically occur in one day in association with enteric viral infection (e.g., rotavirus or norovirus) without hypoglycemia, electrolyte imbalances, or abnormal cerebrospinal fluid [14, 15]. However, a high serum uric acid level has been reported [15, 16]. Brain MRI and EEG are normal in most patients [14, 15], but reversible splenial lesions have been noted in some patients with CwG [17, 18]. Seizures have a good prognosis and require only transient acute antiseizure medication in some patients [14, 15]. CwG is associated with situation-related seizures and rarely with the development of epilepsy. This condition can recur as afebrile seizures associated with acute gastroenteritis in approximately 5% of cases [19].

Cloete and colleagues have summarized the characteristics of pediatric seizures during the Omicron variant wave in 25 children admitted to the hospital [10]. Twenty-one patients (84%) had uncomplicated seizures without copathology (e.g., epilepsy, cerebral palsy, hypoglycemia, or electrolyte imbalances) [10]. Among these, 17 patients (81%) had simple febrile seizures [10]. Four patients were out of the typical age range for febrile convulsions [10]. Although mild clinical impairments have been reported in children with seizures during the Omicron variant surge, most seizures with fever were benign without neurologic deficits compared with the findings of previous reports published before the outbreak [3, 8–13].

There are limited brain MRI data from patients with COVID-19 and neurologic symptoms, even adults. Splenial lesions have been reported in four children who presented with encephalopathy associated with COVID-19 pediatric multisystem inflammatory syndrome; however, none of these patients had seizures [5]. Reversible splenial lesions can be observed in diverse diseases: infections with various pathogens including viruses, seizures, systemic illnesses such as Kawasaki disease or systemic lupus erythematosus, malnutrition, intoxication (e.g., CO or metronidazole), and mountain sickness [20]. Reversible splenial lesions may be caused by intra-myelin edema due to inflammation; these typically resolve within a week [5, 20].

The pathophysiologic mechanism explaining the occurrence of seizures in patients with COVID-19 has not been clearly elucidated. However, there are some hypotheses for the occurrence of neurologic symptoms: (1) direct neuronal injury by viral invasion through the angiotensin-converting enzyme 2 (ACE2) receptor on the neuronal membrane; (2) prothrombotic state triggered by vascular endothelial injury through the ACE2 receptor on endothelial cells; and (3) postinfectious or parainfectious hyperinflammatory state, disrupting the blood-brain barrier *via* immune system dysregulation (e.g., cytokine storm, immune cell activation, and autoantibodies to the nervous system) [6, 7].

New-onset afebrile seizures associated with COVID-19 in previously healthy children without a family history of seizures have been rarely reported [4, 11, 13]. Here, we reported cases of two young children (< 3 years) with COVID-19 who showed new-onset brief and recurrent afebrile convulsions immediately after resolution of a fever. One of them had a reversible splenial lesion, which have rarely been reported in patients with COVID-19, particularly in those without encephalopathy. The clinical characteristics in these patients were similar to those reported in patients with CwG. Therefore, if a patient is not critically ill and remains alert between seizures, in contrast to seizures associated with encephalopathy/encephalitis, long-term antiseizure medication is not recommended.

## Data Availability

Not applicable for our study.

## Abbreviations

COVID-19: Children with coronavirus disease 2019
CwG: Benign infantile convulsions with mild gastroenteritis
ER: Emergency room
PCR: Polymerase chain reaction
EKG: Electrocardiography
MRI: Magnetic resonance imaging
EEG: Electroencephalography
ACE2: Angiotensin-converting enzyme 2
TC: Tonic-clonic
T: Tonic
WBC: While blood cell
WNL: Within normal limit
FLAIR: Fluid-attenuated inversion recovery
DWI: Diffusion weighted image
